# Festival Dinners and Meetings

**Published:** 1893-07-01

**Authors:** 


### FESTIVAL DINNERS AND MEETINGS.

### ROYAL HOSPITAL FOR DISEASES OF THE CHEST.

The festival dinner of the Royal Hospital for Diseases of the Chest, held at the Whitehall Rooms, Hotel Metropole, on Tuesday, 20th ult., under the presidency of Lord Rothschild, resulted in the addition of £3,043 to the funds of the hospital. Towards this amount Lord Rothschild and Mr. T. A. de la Rue (chairman of the Council) contributed 300 guineas each. Lord Rothschild, in proposing the toast of the evening, said that though there might be differences of opinion as to the necessity for entertainments and festivals for providing the necessary funds for charitable institutions, he thought they were unanimous in believing that of all forms of charity the hospitals were the best, for if they restored a man willing to work to health and strength, they really did far more for him than they could possibly do by simply giving him money. (Applause.) No hospital, perhaps, deserved support more than the Royal Hospital for Diseases of the Chest, and he deeply regretted that at the present time the hospital could not afford to keep open thirty of the beds in the two wards opened by Princess Beatrice.

### THE LONDON HOMŒOPATHIC HOSPITAL.

The foundation-stone of this hospital, in Great Ormond Street, was laid by the Duchess of Teck; Princess May was prevented by slight indisposition from accompanying her. Those present included Lord Ebury (the President), Lady Caird, Lady Victoria Grosvenor, the Hon. Frances Grosvenor, the Hon. Wellesley Grosvenor, the Hon Mrs. Norman Grosvenor, the Hon. Mrs. Algernon Grosvenor, Lady Newton, General Beynon, &c. A guard of honour of 100 men of the Post Office Volunteers received her Royal Highness, and an address was presented by Mr. Alan E. Chambre, Chairman of the Building Committee, in which it was stated that the hospital was inaugurated some 50 years ago through the generosity of the late Dr. Frederick Foster Quin and others to succour the sick poor under medical principles. An unwavering adherence to those principles had been followed by ready help from the public, and the efficient training of nurses had elicited warm recognition from the entire medical profession. The Board of Management were proud of a record of 300,000 patients, with a yearly register of 800 in-patients and 10,000 out-patients. The new building would involve an expenditure of £40,000, £30,000 of which had already been provided, and would contain all the most modern improvements in the effective care of the sick and suffering.

Hymns were sung and a dedication prayer read and the foundation-stone raised, copies of the *Times* and of the forty-second annual report of the hospital being placed beneath. The slab was then fixed, the Duchess spread the mortar, and after the lowering of the stone, declared it to be "well and truly laid." The Benediction was pronounced by the Bishop of Bedford.

### CITY ORTHOPÆDIC HOSPITAL.

The anniversary dinner in aid of this charity took place at the Holborn Restaurant, the Lord Mayor presiding. A number of friends of the hospital were present, including Mr. Justice Bruce, Mr. J. A. Rentoul, M.P., and Major Isaacs. In replying to the toast of "The Houses of Parliament," Mr. Rentoul made an amusing speech. The Lord Mayor appealed for increased help on behalf of the hospital, which was in debt to the amount of £6,000. He hoped its work might not be long hampered on that score. During the evening the Secretary, Mr. E. Darenth, announced subscriptions amounting to nearly £900.

### HOSPITAL FOR SICK CHILDREN, GREAT ORMOND STREET.—OPENING OF THE NEW WING.

On Saturday last the Prince and Princess of Wales, accompanied by the Duchess of Fife and the Princesses Victoria and Maud of Wales, paid a visit to this Children's Hospital, on the occasion of the formal opening of the new wing. A large marquee was erected in the garden, and in this the dedication service took place. A large number of visitors were present, amongst whom were Lord and Lady Aberdare and the Hon. Misses Bruce, Lady Jeune, Dr. Abercrombie, Mr. Justice Kekewich, Dr. and Mrs. Barlow, Mr. and Mrs. Hugh Arbuthnot, Sir Arthur and Miss Birch, Baron Hirsch, Colonel the Hon. Charles and Mrs. Elliot, Sir Saville Crossley, Lady Clarke, Viscount Gort, the Hon. Miss Vereker, Sir William Cameron and Lady Gull, Lord and Lady Kinnaird, and the Hon. Spencer Hylton Jolliffe.

A somewhat unaccountable delay in the arrival of the Royal party, the interval, however, being pleasantly enlivened by strains from the band of the Grenadier Guards, under Lieutenant Dan Godfrey, was presently proved to have been caused by an act of kind thoughtfulness on the part of the Princess of Wales, who had caused toys to be distributed to each child in the wards through which she passed.

On their arrival at the hospital the Prince and Princess were received by the Duke of Fife (the President of the hospital), Mr. Arthur Lucas (Chairman), Mr. John Murray (Vice-Chairman), Mr. John Deacon (Treasurer), Dr. Sturges, and Mr. Edmund Owen, and after signing a "Memorial Record" proceeded through the Alexandra and Helena wards, where the Princess and her daughters were presented with bouquets by the small patients, to the marquee. Here the dedication prayers were read by the Bishop of Rochester, and a hymn sung by the choir of St. George the Martyr, Queen Square.

In a few words the Duke of Fife welcomed their Royal Highnesses, and an address was read by Mr. Arthur Lucas explaining the present position of the hospital. The Prince, in reply, said: "Ladies and Gentlemen,—In the name of the Princess, and in my own, I beg to thank Mr. Lucas for the kind address which he has just read to us I can assure you all that we were only too pleased to come here to-day to renew our acquaintance with this hospital. Some twenty or twenty-one years ago we laid the foundation-stone of the original building, and we are only too glad to open another building to-day. We have been through two wards to-day, and I can only congratulate all who have the management of the hospital on the most excellent order in which we found it. The poor children who were there seemed to be so well cared for, and the medical staff and nurses were doing their duty most efficiently. We most gladly come here to take part in this ceremony, and have done so on the special invitation of our son-in-law, the Duke of Fife, who is your President, and who, with my eldest daughter, takes so deep an interest in the welfare of the institution."

A gold key (the gift of the contractor, Mr. W. J. Mitchell, and beautifully designed and engraved by Mr. Lionel Crichton, silversmith, of Kensington) was presented to the Prince, and then came the prettiest part of the ceremony, the presenting of the purses to the Princess. This was done mainly by children, and the long procession which passed in unbroken stream up the steps on one side of the dais to place the offerings in the Princess's hands and down the other, roused much interest among the spectators. One small boy in sailor's garb was quite too absorbed in the important question of how to make his best bow to remember the still more important matter of delivering up the purse he carried, and would have proceeded to bear it off again had not rescue been at hand. This concluded the day's proceedings, and the Royal party departed, the band playing the National Anthem.

The buildings were charmingly decorated with plants and flowers; some of the wards were quite brilliant with roses, carnations, and maidenhair fern. The new wing has been in occupation for some little time, and is a considerable extension on the original structure. It seems a pity that new buildings, so excellently arranged in other respects, should be deficient in that important department, the nurses' accommodation. But so it is; the nurses' dining-room is a bare, cheerless-looking apartment, and we were sorry to find that some of the nurses are housed in dormitories containing no less than twelve beds, only divided by curtains. This is a defect we trust the authorities will remedy at the earliest possible opportunity.

A great feature in this institution is the chapel, which is well worth a visit, being most beautifully fitted and kept.

We understand the donations and purse collections amounted to £3,500, and we warmly congratulate those who were responsible for the management of Saturday's ceremony on the success which has attended it.

### NATIONAL HOSPITAL FOR THE PARALYSED AND EPILEPTIC.

The biennial festival dinner of the National Hospital, Queen's Square, took place on June 23rd at the Hotel Metropole, the Earl of Dudley presiding on the occasion. The Countess of Dudley, Lord Saye and Sele, the Rev. Canon Duckworth, Dr. Russell Reynolds, Sir James and Lady Crichton Browne, Sir William and Lady MacCormac, the Archdeacon of London, Lord Alwyne Compton, General Campbell, Colonel Porter, the Hon. W. and Mrs. Massey Mainwaring, Mr. W. Angerstein, Dr. Buzzard, Dr. Gowers, Mr. Frank Capel, Dr. Gibbon, Mr. and Mrs. Mitchell, the Rev. and Mrs. Dacre Craven, Dr. Ramskill, and the excellent Secretary (Mr. Burford Rawlings) were amongst the numerous guests.

"The Health of Her Majesty the Queen, the Patron of the Hospital," proposed by Lord Dudley and received with much applause, was followed by the toast of "The Prince and Princess of Wales and the Rest of the Royal Family," also proposed by the Chairman. He spoke with great cordiality of the Royal wedding, and the general satisfaction which it would give to the nation, and of festival dinners as valuable epochs in the annals of hospitals, offering occasions for special appeals to the charitable public. The Hospital for the Paralysed and Epileptic was well named 'The National,' for it supplied a long felt national want—patients from over 1,500 towns had already been received within its wards. It was impossible to overestimate the enormous beneficent possibilities of this hospital, or the amount of medical and surgical knowledge which emanated from it. Everyone must feel special sympathy and interest in those mysterious maladies, classed as nervous diseases, which were often sudden in their onset, requiring prompt scientific treatment. Unfortunately these special diseases increase in these modern days of pressure, and it was incumbent on every one to lose no opportunity of supporting an institution for their treatment. The National Hospital, with its handsome and extensive buildings, could not be said to be in any immediate want, but it needed increasing and regular support to enable it to continue its good work—a good machine should be lubricated ungrudgingly.

The absence of the Duke of Westminster, the President of the Hospital, was regretted, but Canon Duckworth expressed the opinion of all in the room when he told Lord Dudley that he considered his diffidence misplaced. He certainly fulfilled his duties as Chairman admirably, and his appeal for the hospital was so eloquent and sympathetic that the cause for which he pleaded could not fail to be benefited by his advocacy.

Speeches were made during the evening by the Hon. W. Massey Mainwaring, General Campbell, Canon Duckworth, Dr. Russell Reynolds, Lord Saye and Sele, Colonel Porter, Chairman of the Board, Sir J. Crichton Browne, and Dr. Buzzard.

The collection made in the room amounted to £1,595, including 126 new annual subscriptions.

The terrible naval calamity, of which tidings had reached London on the day of the dinner, was mentioned by several speakers, and Lord Dudley's simple reference to the presence on the doomed ship of his brother, a midshipman, and of the intention of himself and family to give to the National Hospital £1,000 as a thankoffering, formed an interesting conclusion to a very pleasant festival. More than one of the guests agreed with the comment, "I clean forgot he was a grand lord when he stood there speaking of his own little brother, with his voice shaking just for all the world like a plain, feeling, working man."

Mr. Burford Rawlings is to be congratulated on the excellence of the arrangements, and also on account of the pleasant tribute piad by one or two speakers to his administration of a hospital which possesses such a famous staff of celebrated doctors. The specially kind reference made by Dr. Buzzard to the ability and devotion of the resident medical staff must also be recorded.

Tenders for the construction of a new ambulance conveyance have been received from Mr. Fisher, of Hanley, for the Hanley, Stoke, and Fenton Joint Hospital Board.

Bognor Infirmary.—Alterations in connection with the hot water and heating apparatus have been effected at this establishment, the cost of which amounted to £1,655. Mr. G. E. Vernon-Inkpen, architect, designing the new works.

Hospital at Stornoway.—This is a new hospital, available for the whole population of the Island of Lewis (Scotland), the plans of which were designed by Mr. John H. Gall, architect, of Inverness. These plans, which are to be carried out immediately, show an arrangement for two wards to accommodate six patients each, separated by an administrative department embracing the Matron's room, doctor's room, central hall and vestibule, and two night nurses' duty-rooms, behind which is the surgery and operating rooms, and the kitchen and kitchen offices. The laundry, washing house, and mortuary are contained in a separate block. Above the administrative department are bed-rooms for the nurses and kitchen staff, and a private or separation ward with bath-room and other conveniences. Off each ward is an annexe, containing lavatories and bathrooms for the patients; and the whole arrangement has been so planned as to lend itself to further extension at a later date if required.

